# Recent Advances in Electrochemical Aptasensors for Food Safety Detection

**DOI:** 10.3390/foods15214008

**Published:** 2026-11-09

**Authors:** Jingjing Cui, Jing Cao, Mingxiu Che, Wenjing Zhao, Wenjie Chen, Yibo Song, Mingfei Pan

**Affiliations:** 1 Key Laboratory of Food Quality and Health of Tianjin, Tianjin University of Science and Technology, Tianjin 300457, China; 2 State Key Laboratory of Food Nutrition and Safety, Tianjin University of Science & Technology, Tianjin 300457, China; 3 Shandong Institute for Food and Drug Control, Jinan 250101, China; 4 Administration of Market Supervision Evaluation and Inspection Center, Hohhot 010000, China

**Keywords:** electrochemical aptasensor, sensing strategy, aptamer immobilization, signal amplification strategy, food safety

## Abstract

Food safety detection is an important technical support for protecting public health and maintaining social stability. Owing to the strong affinity and specific recognition ability of aptamers, together with the high sensitivity, rapid response, and easy integration into miniaturized devices offered by electrochemical detection, electrochemical aptamer-based sensors (EAB) have shown promising potential for detecting food contaminants. This review systematically summarizes recent progress in this sensing technology for food safety analysis. It also outlines major aptamer immobilization strategies and discusses electrode materials employed for electrode surface modification. Particular attention is given to their applications in the detection of various contaminants in food safety. Based on current studies, the future development directions and application potential of this technology are also discussed.

## 1. Introduction

Food safety is of great importance to public health, social stability, and economic development. With the rapid growth and increasing complexity of the food supply chain, food products are becoming more vulnerable to contamination from various hazardous substances. Pesticide and veterinary drug residues, mycotoxins, heavy metal ions, and foodborne pathogens may be introduced into foods during different stages of production, processing, storage, and distribution. These contaminants accumulate in the human body through dietary intake and may cause significant adverse effects on human health [1,2]. Conventional analytical methods such as chromatography [3], mass spectrometry [4], and surface-enhanced Raman spectroscopy [5] have been widely employed because of their excellent sensitivity and selectivity. However, they generally require complicated experimental procedures, long analysis times, expensive instruments, and skilled operators. These limitations make it difficult for them to fully meet the growing demand for rapid and on-site detection in modern food safety supervision [6]. Therefore, the development of portable, economical, highly sensitive, and user-friendly sensing technologies is essential for the rapid detection of trace contaminants in complex food matrices.

Biosensing platforms have become an attractive approach for addressing this need, converting molecular recognition events into measurable signals through various transduction mechanisms. Major biosensing transduction modalities include electrochemical (voltammetric, impedimetric, potentiometric), optical (SERS, SPR, fluorescence, colorimetric), and piezoelectric (QCM, surface acoustic wave) approaches, among others, with each method exhibiting specific strengths and drawbacks [7,8,9,10,11]. Optical methods generally provide high sensitivity, rich information content, multiple detection principles, and intuitive readouts, while piezoelectric sensors enable label-free, real-time detection of mass changes at the sensor surface with high mass sensitivity [12]. However, these methods typically require complex optical components, dedicated light sources, or highly accurate frequency-measurement devices, which increase system complexity and operational costs while limiting portability and device integration [13]. In contrast, electrochemical sensors have gained considerable interest in food safety analysis due to their rapid response, high sensitivity, relatively low cost, and strong suitability for miniaturized and on-site applications [13,14].

The performance of electrochemical biosensors is largely determined by the molecular recognition element, which directly affects sensor specificity and selectivity. Electrochemical biosensors integrate the advantages of electrochemical detection with the excellent specificity provided by biological recognition elements. They transform biochemical recognition events into measurable electrical signals, such as current, potential, or impedance, thereby enabling the qualitative and quantitative determination of target analytes and demonstrating great application potential [15]. A typical electrochemical biosensor mainly consists of a transducer, a molecular recognition element, and the target analyte [16]. Among these, the molecular recognition element is a key factor responsible for sensor specificity and selectivity. Common recognition elements include antibodies, enzymes and nucleic acids. However, some traditional biorecognition elements such as antibodies and enzymes still face challenges including poor stability, high production costs, and demanding storage conditions, which may limit their practical application in routine food analysis [17,18]. Among various recognition elements, aptamers have received increasing attention owing to their excellent recognition ability and tunable properties. Aptamers are single-stranded DNA or RNA molecules obtained through the systematic evolution of ligands by exponential enrichment (SELEX) process. They can form specific three-dimensional structures, including hairpins, stem-loops, and G-quadruplexes, which enable them to specifically bind to target molecules through hydrogen bonding, electrostatic interactions, van der Waals forces, π–π stacking, and structural complementarity [19,20,21]. This structure-based recognition mechanism resembles antigen–antibody interactions; therefore, aptamers are commonly known as “chemical antibodies”. Due to their chemical nature, aptamers can be readily synthesized and chemically modified with different functional groups, such as thiol amino, or biotin for efficient immobilization on electrode surfaces [22,23,24,25]. These features make aptamers particularly attractive as recognition elements for electrochemical sensing platforms.

Electrochemical aptamer-based sensors (EABs) combine aptamers as specific recognition elements with electrochemical transducers to translate target-binding events into detectable electrical signals. Upon target recognition, aptamers experience structural rearrangements that influence interfacial electron transfer processes, steric effects, or local charge distribution, thereby enabling quantitative detection of target analytes [26,27]. Importantly, the distinct merit of EABs lies in synergistically integrating the superior molecular recognition capability of aptamers with the inherent advantages of electrochemical transduction. Specifically, aptamers offer high chemical stability, low cost, reproducible synthesis, and facile chemical modification, while electrochemical transduction provides high sensitivity, rapid response, low reagent consumption, cost-effective instrumentation, and easy miniaturization into portable devices without requiring complex optical components [28,29]. Driven by these combined merits, recent technological advances in EABs have rapidly emerged across sensing modes, aptamer immobilization strategies, electrode interface engineering, signal amplification, and real-sample applications, making them increasingly promising for food safety detection. Nevertheless, the practical application of EABs in complex food matrices remains challenging due to limitations associated with aptamer selection efficiency and the antifouling ability of sensing interfaces [30,31]. This review systematically summarizes recent advances in EAB strategies for food safety detection (Graphic Abstract). First, the main sensing strategies, including potentiometric, voltammetric, and impedimetric modes, are introduced and discussed. Second, the immobilization strategies of aptamers on electrode surfaces and the effects of modification materials on electrochemical performance are reviewed in detail. In addition, recent applications of EABs for monitoring different food contaminants are summarized. Finally, current challenges and future perspectives of this field are discussed.

## 2. Electrochemical Aptasensing Strategies

**Figure 1:**
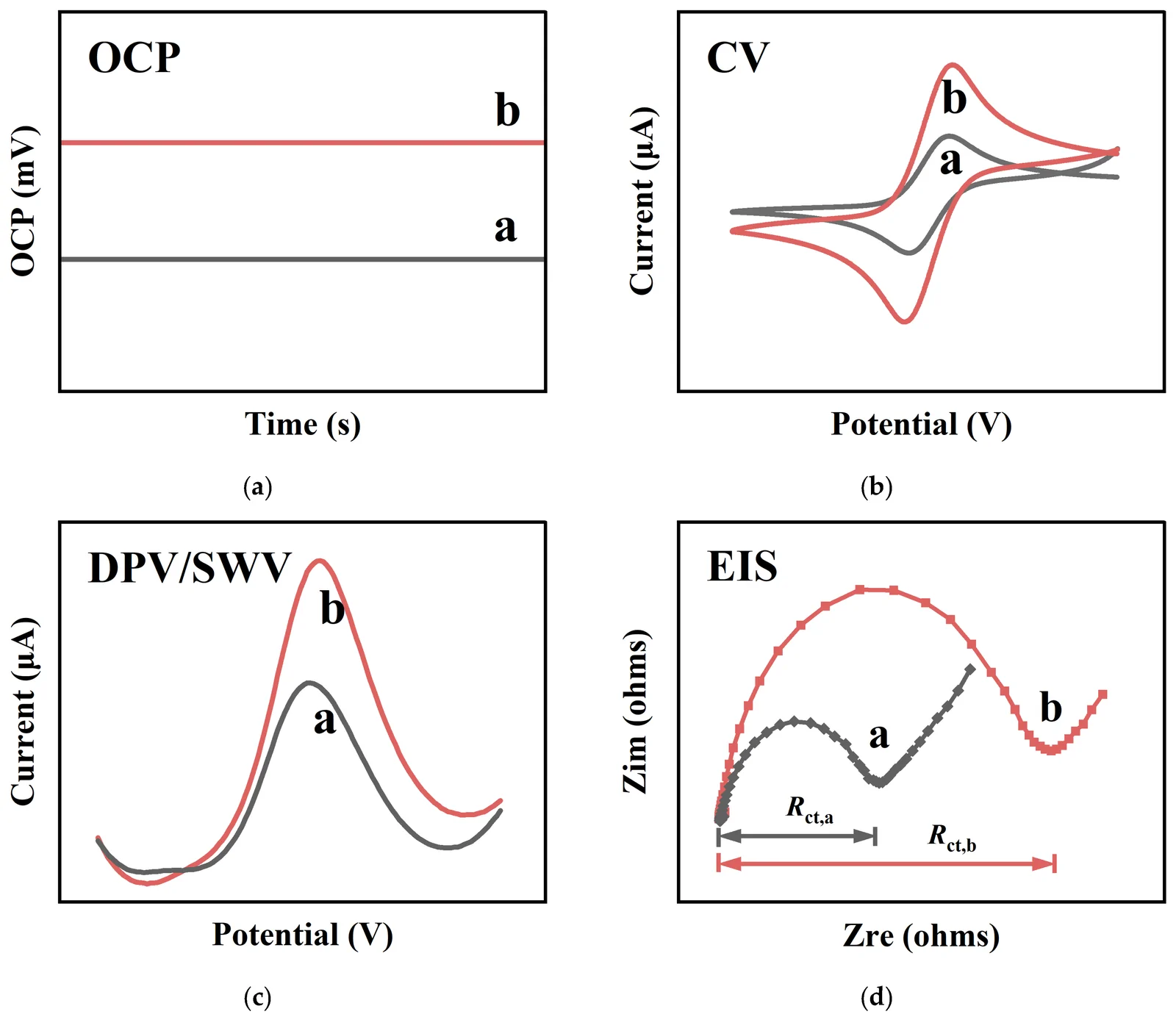
Schematic illustration of various detection modes of electrochemical aptamer-based sensors. (**a**) Open-circuit potential (OCP), where target recognition induces a potential shift (ΔOCP) measured between the working electrode (WE) and reference electrode (RE). (**b**) Cyclic voltammetry (CV) and (**c**) differential pulse voltammetry/square-wave voltammetry (DPV/SWV), where target binding causes variations in peak current (Δ*I*_p_). (**d**) Electrochemical impedance spectroscopy (EIS), where the target-induced interfacial change is reflected by the charge-transfer resistance (*R*_ct_), corresponding to the diameter of the semicircle in the Nyquist plot. Voltammetric and impedimetric measurements are typically performed using a three-electrode system consisting of a working electrode (WE), a reference electrode (RE), and a counter electrode (CE).

EAB have demonstrated their effectiveness as analytical tools for the detection of food-related hazardous substances. The core sensing system usually consists of a three-electrode configuration and an electrolyte solution. The three-electrode system includes a working electrode (WE), counter electrode (CE), and reference electrode (RE), which perform different functions during electrochemical signal generation and acquisition. The WE is the main site for redox reactions of target analytes and interfacial electron transfer, and its surface is commonly modified with functional materials, such as metal and carbon-based nanomaterials, to enhance electrocatalytic activity, electrical conductivity, and interfacial electron transfer efficiency. The CE is generally fabricated using inert conductive materials, such as platinum and graphite, and forms a complete current pathway with the WE to maintain charge balance within the system. The RE provides a stable potential reference, allowing accurate regulation of the potential applied to the WE. Electrochemical measurements are commonly performed in PBS buffer or electrolyte systems containing redox probes, such as Fe(CN)_6_^3−/4−^ with KCl as the supporting electrolyte. As an ion transport medium, the electrolyte can reduce solution resistance, maintain stable ionic strength, and minimize signal interference caused by ion migration, thereby ensuring stable electrochemical reactions and reliable signal acquisition [32]. In the presence of analytes, binding-induced signals are generated and converted into corresponding electrochemical responses, which can be detected within minutes with high sensitivity. According to their working principles, EAB can be classified into potentiometric and non-potentiometric categories. Potentiometric sensors detect changes in electric potential (voltage) at the electrode interface under zero-current conditions. Non-potentiometric sensors include all other electrochemical sensing modes, where the analytical signals are mainly represented by current or impedance changes. They mainly include voltammetric sensors (which monitor current variations using techniques such as CV, DPV, and SWV) and impedimetric sensors (which measure impedance changes, such as EIS) [32,33]. The response profiles of these various detection approaches are illustrated and summarized in Figure 1.

### 2.1. Potentiometric Aptasensor

Potentiometric sensors usually operate under zero-current or open-circuit conditions. Most solid-state potentiometric aptasensors reported for food analysis adopt a two-electrode setup, which consists only of a working electrode (WE, the aptamer-modified sensing electrode) and a reference electrode (RE). In this configuration, the potential difference between the working and the reference electrodes is correlated with the concentration of the target analyte [34]. Based on the sensing interface composition, potentiometric sensors can be divided into polymeric liquid-membrane sensors and solid-state sensors [35]. In contrast, Traditional liquid-membrane potentiometric sensors rely on ionophores for recognition and are mainly suitable for small charged species but show limited ability for non-ionic or macromolecular targets [36].

In contrast, in solid-state sensing configurations, aptamers are immobilized on the surface of conductive substrates. With the ion-to-electron transduction capability of the system, specific interactions between aptamers and targets can generate measurable potential changes [37]. Because no additional labels or complex signal probes are required, this type of sensor has the advantages of simple instrumentation, convenient operation, and easy miniaturization, and has been employed for the detection of various analytes related to food-safety concerns [38,39]. Recognition can be coupled to target recycling and portable readout. Yao et al. [40] reported a flexible graphene paper-based potentiometric aptasensor for kanamycin detection. Graphene paper served as both the conductive substrate and the aptamer immobilization surface, while DNase I-assisted target recycling amplified the recognition-triggered response. Measurements employed a two-electrode configuration with an aptamer-modified graphene paper electrode and a saturated Ag/AgCl reference electrode containing 3.3 M KCl, in 20 mM Tris buffer. Integration with wireless readout demonstrated the potential to combine molecular amplification with portable instrumentation. Direct conformational transduction provides one route to this coupling. Tang et al. [41] developed a label-free potentiometric platform for Pb^2+^ detection using a dendritic gold-modified gold electrode. Pb^2+^ binding induced G-quadruplex formation and altered the interfacial potential, yielding a detection limit of 8.5 pM. The platform was applied to water samples. This example illustrates how a target-dependent structural transition can generate a potentiometric response without an electroactive label. Interfacial charge engineering offers another approach to strengthening recognition-induced responses. Liu et al. [42] constructed a polycation-assisted recognition interface on a screen-printed carbon electrode (SPCE). The positively charged polyethyleneimine (PEI) layer increased aptamer loading and enhanced target-induced interfacial charge changes. Open-circuit potential measurements were performed against an Ag/AgCl reference electrode containing 3.0 M KCl, using 0.05 M Tris-HCl buffer at pH 7.4 as the measurement medium. The sensor achieved a detection limit of 3.43 pM and recoveries of 88.53–104.25% in spiked milk samples. Here, the interface contributed to signal generation by regulating the local charge environment surrounding the recognition layer.

Potentiometric aptasensors have advanced substantially, yet their practical use is still restricted by several factors, including relatively small potential shifts, insufficient interfacial stability and reproducibility, limited aptamer regeneration, and matrix-induced potential drift [43,44]. Reliable food analysis therefore requires stable recognition interfaces and effective control of matrix-induced potential drift, alongside signal amplification and device integration [33,45].

### 2.2. Non-Potentiometric Aptasensors

#### 2.2.1. Voltammetric Aptasensor

Voltammetric aptasensors quantify targets by measuring recognition-induced changes in the current of an electroactive reporter. Because aptamers and many food contaminants do not provide suitable intrinsic redox signals, reporters such as methylene blue (MB), ferrocene (Fc), and [Fe(CN)_6_]^3−/4^ are commonly employed [46]. Voltammetric aptasensors typically involve three consecutive processes, namely target recognition, signal transduction, and electrochemical readout. Cyclic voltammetry (CV), differential pulse voltammetry (DPV), and square wave voltammetry (SWV) mainly serve as readout techniques to monitor changes in electrochemical-probe signals triggered by molecular recognition. By coupling aptamer recognition with voltammetric measurement, such sensors can rapidly screen and quantify trace contaminants, including pesticides, heavy metals, and biotoxins, in complex food matrices, showing great promise for food safety monitoring and on-site detection [47,48].

CV is useful for assessing electrode modification and interfacial electron transfer, but it can also provide the analytical readout for recognition-controlled reporter redistribution [49]. Owing to its simple operation and rapid response, CV has become a commonly used characterization tool for verifying interfacial construction and screening material performance in electrochemical sensing systems. As an example of redox-probe-mediated signal transduction, Taghdisi et al. [50] developed an aptasensor for acetamiprid (ACE) using MB as the electroactive probe and exonuclease I (Exo I) for signal amplification. ACE binding disrupted aptamer–complementary strand hybridization, releasing MB and increasing the CV peak current. Exo I digestion of the free complementary strand further reduced MB binding and increased reporter release. Thus, the analytical signal arose from target-dependent nucleic acid dissociation coupled with enzymatic amplification. Reporter loading and conductive interfaces can further enhance voltammetric responses. Du et al. [51] constructed a Pb^2+^ aptasensor on an SPCE using a graphene oxide/polyamidoamine-gold (GO/PAMAM-Au) composite interface and an MB-gold nanoparticle-single-stranded DNA probe. Incorporating the reporter-bearing probe increased the CV response 1.41-fold. This design illustrates the complementary roles of interface engineering and reporter enrichment: the conductive substrate supports signal collection, while the nucleic acid-associated probe increases the available electrochemical response.

DPV is a pulse-based electrochemical readout technique in which potential pulses are superimposed on a staircase waveform. The current difference obtained from measurements taken before and near the end of each pulse is used as the analytical response. By minimizing capacitive and background contributions, DPV facilitates the resolution of relatively small faradaic signal changes and is therefore widely used for quantitative electrochemical sensing [52]. Li et al. [53] developed a dual-ratiometric aptasensor based on a hairpin–tetrahedral DNA nanostructure (HP-TDN) for simultaneous detection of malathion and profenofos. Target-dependent changes in Pb^2+^- and Cd^2+^-based reporter signals were measured by DPV in PBS at pH 6.0. The design combined a structured DNA scaffold with multiple electrochemical reporters, enabling separate recognition events to be resolved within one sensing platform. Target-induced reporter release provides a different route to DPV signal generation. Bahaabadi et al. [54] employed aptamer-functionalized, ZIF-8-derived nanoporous carbon loaded with MB (CZ@MB) for sulfadimethoxine detection. Target binding induced aptamer conformational changes and MB release, decreasing the DPV oxidation peak current. Measurements were conducted in 0.1 M PBS at pH 7.4 using an Ag/AgCl reference electrode containing saturated KCl. In this configuration, aptamer recognition-controlled reporter retention, directly linking target binding to a signal-off response. Chen et al. [55] enhanced electron transfer at the electrode interface to improve DPV signal response by constructing a highly conductive sensing interface using CuNPs/ZIF-8 composites, achieving ultrasensitive detection of aflatoxin B_1_ (AFB_1_). The sensor exhibited a very low LOD of 1.13 pg/mL and a wide linear range from 10 to 1.0 × 10^6^ pg/mL.

SWV is a pulse electrochemical technique that applies square wave pulses on staircase potentials and collects net current signals from forward and reverse currents. This approach reduces double-layer capacitive interference, improves signal-to-noise ratio, and enables quantitative analysis through the linear relationship between peak current and analyte concentration [56]. Combining high sensitivity with reaction mechanism analysis capability, SWV is widely used for rapid detection of food and environmental contaminants [57]. Conformational switching is a common signal transduction strategy in SWV-based aptasensing. Zhou et al. [58] developed an ACE aptasensor incorporating a triplex DNA molecular switch. ACE recognition disrupted the triplex structure and released a trigger strand, which promoted assembly of G-quadruplex/hemin complexes on the electrode. The resulting increase in the SWV response enabled a detection limit of 4.67 × 10^−3^ nM. Electrochemical measurements employed Fe(CN)_6_^3−/4−^ and PBS media for the corresponding characterization and detection procedures. The sensing mechanism connected aptamer recognition to trigger release and subsequent formation of signal-generating structures. Building on this conformational switching mechanism, Li et al. [59] coupled ACE recognition to a surface-confined primer exchange reaction (PER). Target-induced aptamer rearrangement released a trigger that initiated PER, producing G-rich DNA sequences capable of forming G-quadruplex/hemin structures. Amplification of these structures enhanced the SWV response, yielding a detection limit of 2.73 pM and enabling ACE determination in vegetable, fruit, and soil samples. Compared with direct reporter redistribution, this strategy introduced an additional nucleic acid reaction between recognition and readout, increasing the signal generated from the initial binding event.

Together, these studies illustrate how voltammetric aptasensing extends from recognition-controlled probe redistribution to reporter enrichment, multiple signal channels, and nucleic acid-mediated amplification. The resulting performance depends on the entire recognition–transduction pathway. Direct reporter-release strategies offer relatively simple signal conversion, whereas amplification reactions can strengthen the response at the cost of additional reagents and procedural steps. Likewise, distinguishable reporters support multiplex analysis but require control of signal overlap and interactions between recognition elements. Strategy selection should therefore balance sensitivity with assay complexity, reproducibility, and compatibility with food matrices.

#### 2.2.2. Impedimetric Aptasensor

Unlike voltammetric methods that quantify targets based on variations in current responses, electrochemical impedance spectroscopy (EIS) measurements for aptasensing are mostly performed in a standard three-electrode electrochemical cell to obtain reliable interfacial impedance data. EIS enables label-free quantitative analysis by monitoring variations in interfacial charge-transfer resistance (*R*_ct_) induced by target binding. Faradaic impedimetric aptasensors commonly employ a soluble redox probe, typically [Fe(CN)_6_]^3−/4−^, to reflect electron transfer at the electrode–solution interface. Target binding can alter aptamer conformation, surface charge, steric hindrance, or interfacial packing, thereby tuning redox probe electron transfer kinetics and yielding a measurable *R*_ct_ change [60]. This technique is characterized by a simple setup, high sensitivity, and suitability for in situ, real-time, and label-free detection in complex matrices; accordingly, it has been widely applied in the detection of pathogenic bacteria, pesticides, and heavy-metal ions [61,62,63]. Since *R*_ct_ is highly sensitive to surface binding events, it is employed as the primary readout for label-free impedimetric aptasensors. Capitalizing on this principle, Zhou et al. [64] fabricated an aptasensing platform using cerium-based metal–organic framework and covalent organic framework hybrid nanostructures (Ce-MOF@MCA) for ultrasensitive oxytetracycline (OTC) detection in water. The specific recognition of OTC triggers aptamer binding and G-quadruplex formation, which remodels the electrode interface and modulates the electron-transfer resistance of the [Fe(CN)_6_]^3−/4−^ probe, producing detectable *R*_ct_ variations for ultrasensitive OTC quantification. Hou et al. [65] fabricated a label-free OTA aptasensor with dual signal amplification using a Nafion-stabilized MWCNTs/Au nanopopcorn-modified electrode. Upon ochratoxin A (OTA) recognition, specific aptamer binding induced conformational changes and formed a denser biolayer at the interface, markedly increasing *R*_ct_. This dense layer enhanced steric and electrostatic hindrance to [Fe(CN)_6_]^3−/4−^ approaching the electrode, further restricting interfacial electron transfer and amplifying the impedance response. The sensor achieved a detection limit of 1 pg/mL.

Such label-free impedance-based EABs eliminate labor-intensive electrode-modification procedures. Benefiting from simplicity, low cost and high specificity, they represent a practical and cost-effective sensing strategy for rapid and accurate determination of contaminants in food matrices.

## 3. Aptamer Immobilization Strategies

**Figure 2:**
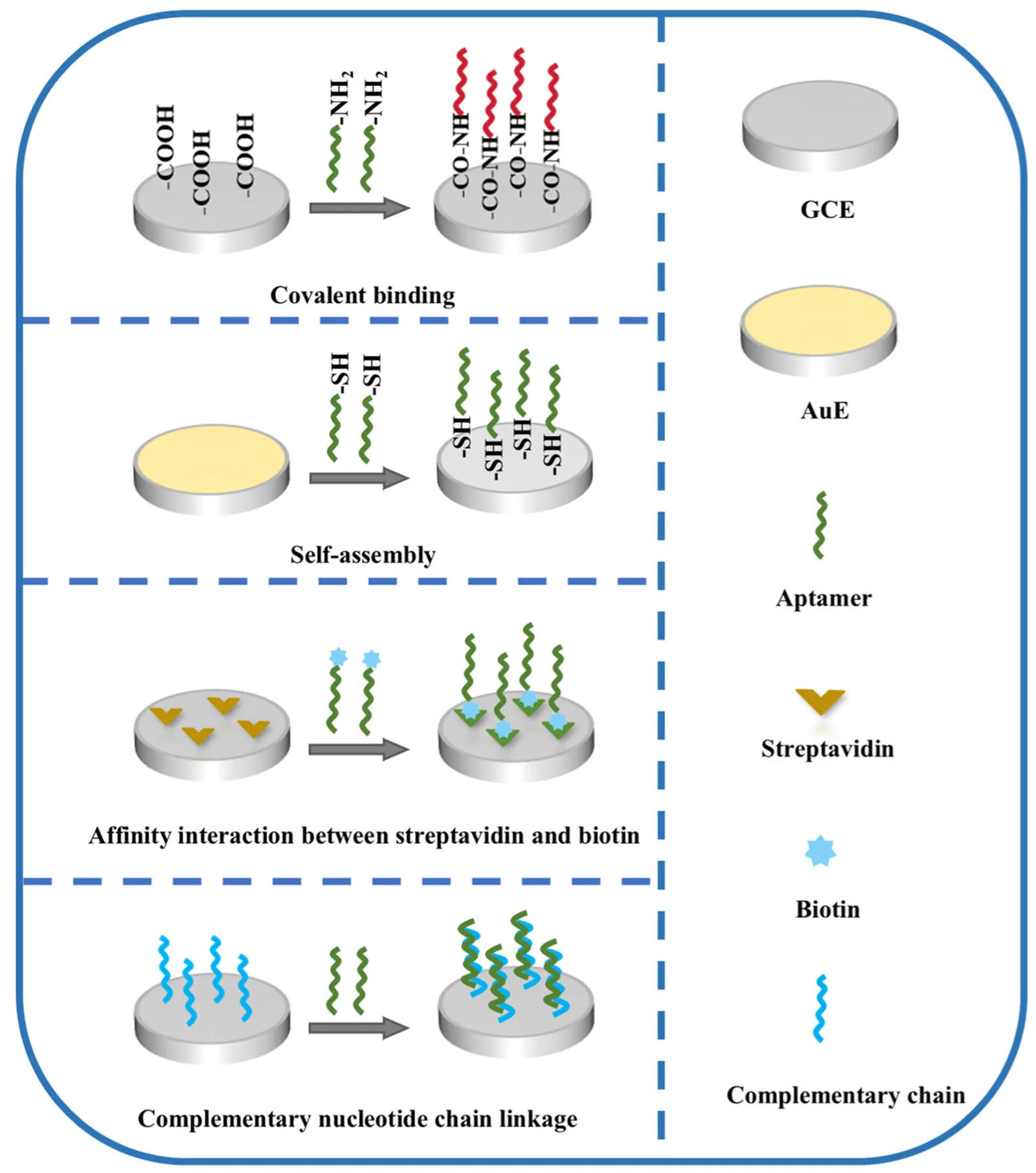
Schematic illustration of different immobilization strategies for aptamers.

The analytical performance of electrochemical aptasensors depends not only on the signal transduction mechanism and electrode conductivity but also on how aptamers are anchored and presented at the electrode interface. Immobilization affects aptamer loading, structural accessibility, target-binding efficiency, interfacial stability, and signal reproducibility. Conventional non-covalent interactions, including hydrogen bonding, π-π stacking, electrostatic interactions, and hydrophobic forces, can facilitate aptamer adsorption but may provide insufficient stability, leading to aptamer desorption and variable sensing responses [66], but the relatively weak interaction forces may result in insufficient stability and poor signal reproducibility. To address these limitations, various strategies have been developed, such as covalent binding [67], self-assembly [68], affinity interaction between streptavidin and biotin [69], and complementary nucleotide chain linkage [70] (Figure 2). The effects of different immobilization approaches on the performance of electrochemical aptasensors are also described (Table 1).

### 3.1. Covalent Binding

Covalent immobilization is achieved by introducing functional groups, such as amino groups (-NH_2_), at the terminal end of aptamers, which react with electrode surface-modified active groups, including carboxyl groups (-COOH) or hydroxyl groups (-OH), to form stable covalent bonds at the electrode interface [71]. This method is commonly based on N-(3-dimethylaminopropyl)-N′-carbodiimide/N-hydroxysuccinimide (EDC/NHS)-mediated amine–carboxyl coupling reactions (-CONH-) as well as azide–alkyne cycloaddition reactions [72,73]. Compared with physisorption, covalent immobilization provides stronger chemical interactions and enhanced structural stability, allowing aptamers to be firmly attached to electrode surfaces and effectively reducing aptamer desorption and nonspecific adsorption [74]. Current developments extend beyond simple chemical anchoring toward the cooperative use of surface chemistry and nanostructured electrode materials.

Khosropour et al. [72] immobilized an amino-modified aptamer on a VS_2_QDs-GNP/CMWCNTs-modified glassy carbon electrode through amide bonding. Combining differential pulse voltammetry (DPV) and electrochemical impedance spectroscopy (EIS), the platform enabled diazinon detection with limits of detection (LODs) of 11 fM and 2 fM, respectively. Maghsoudi et al. [67] developed a carboxyl-rich layer on a gold electrode by diazonium electrografting and subsequently coupled a glyphosate aptamer through EDC/NHS chemistry. The resulting sensor achieved an LOD of 0.67 nM and retained approximately 93.8% of its initial signal after 21 days of storage at 4 °C. An important development is the use of multiple interactions to strengthen aptamer anchoring. Yuan et al. [79] combined glutaraldehyde cross-linking, Au–N coordination, and C=N bond formation to construct a cooperative immobilization network on a nanomaterial-modified electrode. The resulting methamphetamine aptasensor achieved an LOD of 0.87 pg/L and retained 94.44% of its initial signal after 15 days at 4 °C. This approach illustrates how combining different chemical interactions can improve immobilization stability beyond that provided by a single anchoring mechanism.

Despite these advantages, covalent immobilization often involves multistep surface modification. Residual activating or cross-linking reagents may also affect the interface and increase background signals. Further optimization is therefore needed to balance anchoring strength, preservation of aptamer recognition activity, fabrication complexity, and analytical performance [75].

### 3.2. Self-Assembly

Self-assembly immobilization generally involves the introduction of thiol (-SH) groups at the terminal position of aptamers, followed by the formation of strong Au-S coordination bonds between sulfur atoms and gold electrode surfaces [76]. This technique is advantageous in that it does not require complicated electrode pretreatment, is user-friendly, and has become one of the most widely adopted and efficient immobilization strategies in current use. Zhen et al. [80] constructed an electrochemical sensing interface based on a thiol-functionalized aptamer (SH-apt), which was directly assembled onto the AuE surface through Au–S self-assembly interactions, enabling specific recognition of ACE. The sensor achieved a LOD of 1 nM. In the same year, to improve aptamer immobilization efficiency and increase the number of available binding sites, Xu et al. [81] retained the same Au-S self-assembly chemistry but replaced the bare gold electrode with a highly porous gold (HPG)-modified surface fabricated by electrodeposition. The stable immobilization of ACE-apt on the HPG/AuE surface was achieved via Au-S self-assembly. The sensor showed a good linear relationship in the range of 0.5–300 nM, with a LOD as low as 0.34 nM. Further development has involved coupling aptamer immobilization with signal transduction. Ma et al. [82] assembled an ACE aptamer on a multifunctional AuNPs/hBP/TH/PLL/PTh-modified glassy carbon electrode. Target binding induced aptamer folding, exposing additional phosphate backbone sites and promoting the enrichment of the positively charged RuHex probe. This design achieved an LOD of 9.407 fM over a linear range of 10 fM–1 μM. The enhanced response therefore arose not simply from aptamer attachment, but from the coupling of target-induced conformational changes with electrochemical probe accumulation.

Although Au-S interactions enable strong and stable attachment of thiolated aptamers on gold electrode surfaces, exposed electrode areas may cause nonspecific adsorption. Surface-blocking molecules such as MCH and BSA are introduced to occupy unmodified regions and prevent unwanted molecular adsorption, effectively reducing interference and improving detection specificity and reliability [76,83].

### 3.3. Affinity Interaction Between Streptavidin and Biotin

Streptavidin (SA) specifically binds biotin with high affinity, enabling the stable attachment of biotin-labeled aptamers (Bio-apt) onto electrode surfaces through specific biotin–SA recognition interactions. Its multivalent binding capability allows higher aptamer immobilization density, improving sensor stability and sensitivity [77]. Beyond the affinity interaction itself, electrode functionalization and the stability of the SA layer are important determinants of overall sensing performance. Kaur et al. [84] immobilized an ochratoxin A (OTA) aptamer on an indium tin oxide electrode using this strategy. The sensor retained approximately 85% of its initial current response after seven consecutive measurements, while six independently prepared electrodes showed a relative standard deviation (RSD) of 0.45%. Similarly, Hou et al. [27] combined SA–biotin recognition with a Nafion-stabilized, functionalized multiwalled carbon nanotube (f-MWCNT) interface. Carboxyl groups on the nanotubes were activated using EDC/NHS to anchor SA, followed by the attachment of biotinylated OTA aptamers. The sensor achieved an LOD of 1 pg/mL and retained 94.3% of its initial current response after 15 days of storage.

These studies demonstrate that affinity immobilization can provide reproducible aptamer attachment when integrated with a suitably functionalized electrode interface. However, the initial immobilization of SA may require additional covalent coupling steps, increasing fabrication complexity and cost. Further improvements should therefore consider not only binding stability but also simplified preparation, reproducibility, and compatibility with scalable sensor fabrication [78].

### 3.4. Complementary Nucleotide Chain Linkage

The complementary nucleotide strand ligation immobilization method introduces a short complementary sequence at the terminal region of the aptamer and achieves oriented immobilization and stable fixation of aptamers at the electrode interface through specific hybridization between this sequence and a complementary DNA strand (cDNA) pre-functionalized on the electrode surface. This approach does not rely on structural changes of aptamers during target recognition, and it exhibits wide applicability for the construction of different aptamer-based sensing platforms [70]. Lin et al. [85] attached an amino-modified capture strand to a screen-printed carbon electrode through amide coupling and subsequently hybridized a ferrocene-labeled aptamer to the capture strand. The resulting sensor detected Staphylococcus aureus with an LOD of 0.97 CFU/mL and retained more than 97% of its initial signal after 30 days of storage at 4 °C. A more integrated design was reported by Jia et al. [86], in which a complementary DNA strand was assembled on an NC@GC/AuNPs interface through Au–S interactions. The strand served both as an aptamer anchoring element and as a component of signal transduction. Recognition of FIP displaced the aptamer and exposed complementary binding sites; this process was combined with a PtPdCo nanozyme-based MB amplification system and secondary hybridization mediated by a signal DNA strand. The resulting sensor achieved an LOD of 76 pg/mL. This design illustrates how an immobilization component can be incorporated into the signal-conversion pathway rather than functioning solely as a structural anchor. Vit et al. [87] combined molecular docking simulations with aptamer sequence optimization to identify a shortened, high-affinity core sequence for okadaic acid (OA). Improving aptamer structure and recognition-site accessibility enabled specific binding within 5 min. The sensor achieved an LOD of 2.5 nM, with recoveries of 82–103% in mussel samples. This example highlights the complementary roles of aptamer sequence optimization and interface design in improving target recognition.

Nevertheless, hybridization efficiency is sensitive to experimental conditions. Electrostatic repulsion and steric hindrance can restrict strand pairing and reduce immobilization efficiency. Optimization of parameters such as pH and temperature remains important for achieving reliable interfacial assembly [70].

## 4. Electrode Modification Materials

**Figure 3:**
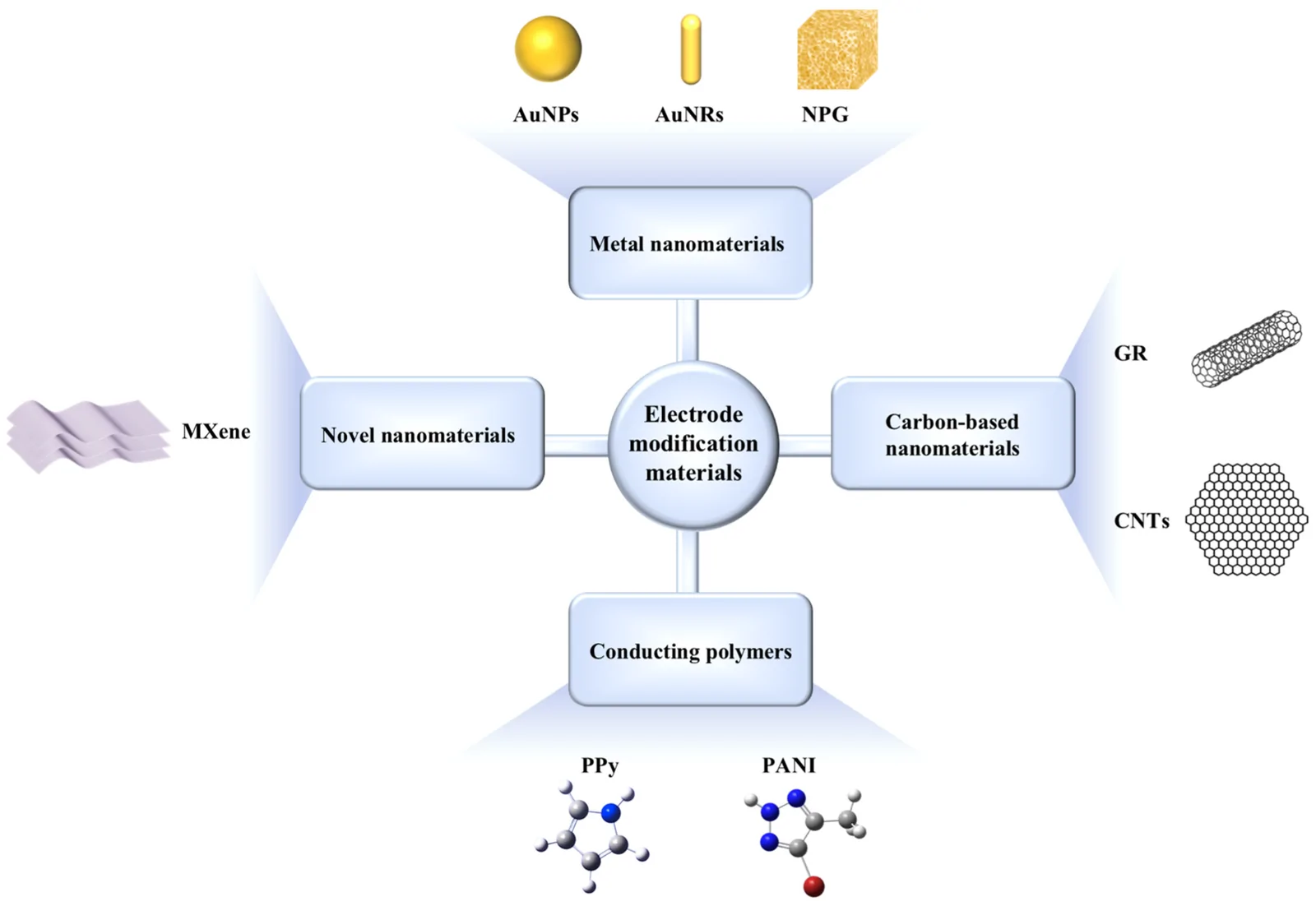
Classification of electrode modification materials.

Electrode modification is an important strategy for improving the performance of electrochemical aptasensors [88,89]. Early studies primarily relied on conventional electrode surfaces and single-component modifiers to improve electrical conductivity and provide sites for aptamer immobilization [90,91]. With the development of nanomaterials, metal nanomaterials [92], carbon-based nanomaterials [27], and conducting polymers [93] have been increasingly employed for electrode modification to increase the electrochemically active surface area, facilitate electron transfer, and improve interfacial functionalization. In addition, emerging two-dimensional materials such as MXenes [94], with their high electrical conductivity and tunable surface terminations, have expanded the possibilities for electrode design. In recent years, research has increasingly focused on nanostructure morphology control, surface chemistry optimization, and the integration of complementary materials to construct multifunctional sensing interfaces (Figure 3).

### 4.1. Metal Nanomaterials

Metal nanomaterials exhibit excellent electrical conductivity, high specific surface area, and abundant surface-active sites, which can significantly facilitate electron transfer at the electrode interface, improve biomolecule immobilization efficiency, and accelerate interfacial reaction kinetics. Therefore, they have been widely used in the interface construction and signal amplification of electrochemical biosensors [89]. Early applications primarily focused on using metal nanoparticles to improve electrode conductivity and provide additional sites for aptamer immobilization. Subsequent studies have increasingly focused on controlling particle size and morphology, constructing high-surface-area structures, and integrating metal nanomaterials with other functional components to enhance signal transduction and detection performance.

Gold-based nanomaterials, including AuNPs, AuNRs, and NPG, are widely used in electrochemical aptasensors to immobilize thiol-modified aptamers through Au–S interactions. Among them, AuNPs can construct conductive interfaces and promote stable nucleic acid attachment [95]. Liu et al. [96] incorporated AuNPs into a bisphenol A (BPA) sensing platform based on a ratiometric signal strategy. The charge-transfer resistance *R*_ct_ decreased from 206.2 Ω at the bare glassy carbon electrode (GCE) to 100.3 Ω after AuNP modification, and the sensor achieved a limit of detection (LOD) of 0.6 pM. This result illustrates the contribution of metal nanostructures to interfacial electron transfer and signal readout. Li et al. [97] extended the application of AuNPs to a screen-printed paper-based sensing platform for the rapid detection of AFB_1_ in peanut kernels and walnut kernels after simple sample extraction. The sensor showed a good linear relationship in the range of 20–1000 ng/mL, with an LOD as low as 7.6 ng/mL, providing a universal detection platform with the advantages of low cost, ease of transport, and precise quantification. Beyond single-component modification, recent designs increasingly combine metal nanomaterials with other functional materials to construct heterointerfaces. Han et al. [95] developed an AuNPs/reduced MoS_2_ interface using thionine and 6-ferrocenylhexanethiol as dual signal probes for the simultaneous detection of zearalenone (ZEN) and fumonisin B_1_ (FB_1_) in maize. Compared with the MoS_2_-Au substrate, the reduced MoS_2_–Au interface achieved an LOD of 5 × 10^−4^ ng/mL, representing an approximately tenfold improvement. This approach illustrates how combining conductive metal nanostructures with a complementary material can improve the overall sensing response.

Changes in nanostructure geometry provide another route to performance enhancement. AuNRs offer anisotropic structures that can support interfacial electron transfer and surface functionalization. Gold nanorods (AuNRs) can support interfacial electron transfer and surface functionalization, and their high conductivity facilitates electron transfer at the electrode interface, making them promising candidates for electrochemical aptasensors [98]. Based on these advantages, Jiang et al. [99] developed a vertically assembled AuNR-modified screen-printed electrode for mercury-ion detection. The modified electrode exhibited an *R*_ct_ of 75 Ω, compared with 837 Ω for the bare electrode, and achieved an LOD of 0.3 pM, which falls well below the maximum contaminant level of Hg^2+^ in drinking water set by the U.S. Singh et al. [100] used gold nanorods on a fluorine-doped tin oxide electrode, increasing the electrochemically active surface area from 0.7 to 2.09 cm^2^ for the detection of staphylococcal enterotoxin A. These results indicate that metal nanostructure design can improve sensing performance by increasing the accessible electroactive area and facilitating interfacial charge transfer. Three-dimensional porous structures provide a further development beyond conventional particulate modifiers. NPG combines electrical conductivity with a porous architecture that offers additional surface area for aptamer immobilization [101]. Wang et al. [102] constructed an NPG-based aptasensor for ochratoxin A (OTA) using target-induced complementary-strand displacement. The sensor achieved an LOD of 0.4 pg/mL over a linear range of 1 pg/mL–2 ng/mL.

Overall, metal nanomaterials have evolved from conductive additives into tunable interfacial components that support aptamer immobilization, signal enhancement, and portable sensor construction. Nevertheless, noble-metal cost, nanoparticle aggregation, and fabrication complexity remain important limitations. Future developments should focus on controlling particle distribution, reducing material consumption, improving the stability of metal–support interfaces, and integrating metal nanostructures with other functional materials.

### 4.2. Carbon-Based Nanomaterials

Carbon-based nanomaterials are functional materials composed of carbon frameworks, including 0D nanodiamonds and carbon quantum dots, 1D carbon nanotubes and carbon nanohorns, 2D graphene, and 3D mesoporous carbon structures [103]. Their high specific surface area, excellent conductivity, and biocompatibility make them attractive for EAB construction. Furthermore, surface oxygen-containing groups (e.g., hydroxyl and carboxyl groups) enable the covalent coupling of aptamers and ordered immobilization of probes. Additionally, the high specific surface area enables a higher aptamer loading density, which in turn improves sensor recognition efficiency and signal transduction performance. Among them, GR and CNTs are commonly used for constructing high-performance EAB interfaces due to their excellent electron mobility and stable interfacial properties [104].

Graphene (GR) is a two-dimensional sp^2^ carbon material with excellent conductivity, high carrier mobility, and high specific surface area, showing great potential for electrochemical sensing applications. However, strong π-π stacking and van der Waals interactions between GR sheets induce aggregation. This reduces the effective surface area and electron transfer efficiency, thereby limiting sensor performance [105]. To address the aggregation issue of graphene, graphene oxide (GO) and reduced graphene oxide (rGO) have been developed through oxidation–reduction strategies. rGO partially restores the sp^2^ conjugated network while retaining oxygen-containing functional groups, offering high conductivity, high specific surface area, electrochemical stability, and biocompatibility. It also suppresses aggregation and enables high aptamer loading with controllable immobilization, making it a promising material for high-performance EAB interfaces [106].

On this basis, Yadav et al. [22] constructed an EAB using rGO/SPGE as the sensing substrate for the detection of botulinum neurotoxin A (*BoNT/A*), with a linear range of 1–100 pM and an LOD as low as 1 pM. To further improve the sensitivity of rGO-based sensors, Wang et al. [107] electrodeposited AuNPs onto a rGO-modified screen-printed electrode for the quantitative detection of thiamethoxam (TMX). In this system, AuNPs/rGO/SPE exhibited a low *R*_ct_ of 1.91 × 10^−6^ Ω, much lower than that of bare GCE and rGO/SPE, indicating enhanced electron transfer. The sensor achieved an 18.14% signal change for TMX, about 6.25-fold higher than the bare electrode, demonstrating improved sensing performance. Amini-Nogorani et al. [108] introduced a Cu-Ni-BTC-MOF into a rGO-based composite interface to construct a ternary sensing platform. EIS results showed decreased semicircle diameter and *R*_ct_ of rGO-Cu-Ni-BTC-MOF/GCE, attributed to the synergistic effect of rGO’s high conductivity and the MOF’s large specific surface area. The sensor achieved a linear range of 1.0 × 10^−3^–300 ng/mL and an LOD of 4.6 × 10^−4^ ng/mL for OTA detection in barley flour.

Carbon nanotubes (CNTs) are one-dimensional tubular nanomaterials formed by rolled-up graphene sheets, including single-walled carbon nanotubes (SWCNTs) and multi-walled carbon nanotubes (MWCNTs). MWCNTs possess a high specific surface area, excellent conductivity, and efficient electron transport. Via covalent and non-covalent functionalization, MWCNTs enable efficient immobilization of aptamers, improving the sensitivity and stability of EABs [109]. Shen et al. [110] constructed an Apt/MCH/cDNA/PDA/MXene/MWCNTs/NiCo_2_O_4_/SGPGE sensing platform using a layer-by-layer assembly strategy for AFB_1_ detection, achieving an LOD as low as 1.89 ng/mL. Nitrogen doping and nanoparticle incorporation provide further approaches for tuning interfacial properties, Li et al. [104] developed an N-doped MWCNTs/AgNPs modified electrode for the detection of Pseudomonas aeruginosa (P. aeruginosa). The conductive network of N-MWCNTs and the electric field enhancement of AgNPs synergistically improved electrode activity. The *R*_ct_ of N-MWCNTs/AgNPs/SPCE was reduced to 212.3 Ω compared with that of the bare SPCE (909.9 Ω). The sensor showed a linear range of 10^−1^–10^6^ CFU/mL with an LOD of 0.0798 CFU/mL.

Carbon-based nanomaterials tend to aggregate due to π-π interactions and van der Waals forces, reducing dispersion stability and charge transfer efficiency. Surface functionalization strategies, including covalent grafting, non-covalent modification, and composite construction, can suppress aggregation by introducing functional groups or integrating other materials. These approaches improve dispersion, conductivity, and interfacial stability, facilitating their application in electrochemical sensors.

### 4.3. Conducting Polymers

Conducting polymers (CPs) have attracted extensive attention because their electropolymerized films can simultaneously serve as charge transfer interfaces and aptamer immobilization carriers. In addition, the thickness, morphology, and surface chemistry of CP films are tunable, which facilitates the construction of stable sensing interfaces and improves aptamer loading efficiency. These features promote signal transduction, enhance analytical performance and sensor stability, and thus enable wide use in the construction of electrochemical aptasensors [111,112]. CPs mainly include polyaniline (PANI), polypyrrole (PPy), and polythiophene (PTh). Among them, PPy and PANI are the most extensively studied and representative materials. These polymers are typically synthesized from aniline or pyrrole monomers through chemical oxidative polymerization or electropolymerization. Their electrical conductivity originates from delocalized π-electrons, which enable charge transport along the conjugated polymer backbone. Early studies primarily focused on optimizing the performance of single-component systems. The electrical and electrochemical properties of CPs depend on monomer selection, polymerization conditions, doping, and film morphology. Das et al. [113] showed that optimizing the oxidant-to-monomer ratio during PANI synthesis could improve its capacitive behavior and charge-transfer properties. However, a single polymer component may not simultaneously provide high conductivity, sufficient active sites, and a stable interface. These limitations have motivated the development of polymer–nanomaterial composites. Bahrami et al. [114] synthesized polypyrrole (PPy)/multiwalled carbon nanotube (MWCNT) composites through electrochemical polymerization. In this system, PPy was deposited onto the MWCNT surfaces, forming a polymer-coated nanotube structure, while MWCNTs acted as conductive bridges connecting PPy domains and facilitating charge transport within the composite. The incorporation of MWCNTs established conductive pathways within the polymer matrix, thereby enhancing the electrical conductivity of the composite films. Hamami et al. [115] sequentially modified SPCE with MoS_2_ and electropolymerized PPy nanoparticles onto its surface. The synergistic charge transfer between the two materials reduced *R*_ct_ to 20 Ω, far lower than that of MoS_2_ alone (412 Ω). After covalent immobilization of naphthoquinone and AMP aptamer via EDC/NHS chemistry, the sensor achieved an LOD of 10 pg/L for AMP, outperforming MoS_2_-only (66 pg/L) and PPy-only (71 pg/L) systems, demonstrating the synergistic role of PPy/nanomaterial composites in enhancing sensing performance. Wang et al. [116] further developed a three-dimensional interconnected PPy/NiFe layered double hydroxide (LDH)/Ti_3_C_2_ MXene network for organophosphorus pesticide detection. This architecture combined the film-forming and bridging properties of PPy with the complementary functions of LDH and MXene, illustrating the progression from single-polymer modification to interconnected multifunctional networks. Similar improvements have been reported in other polymer-based composite systems. Vaeli et al. [93] prepared a poly(o-aminobenzoic acid-co-aniline) layer on graphite sheets by electrochemical copolymerization. The composite reduced *R*_ct_ from 567.9 Ω to 16.6 Ω and enabled tetracycline detection with an LOD of 3.3 × 10^−17^ M in milk, egg, and honey samples.

These studies indicate that the development of conducting polymer-based aptasensors increasingly relies on interactions between polymers and nanomaterials, as well as the construction of continuous conductive interfaces. However, issues such as polymer film thickness, fabrication reproducibility, long-term stability, and nonspecific adsorption still require further attention. Future research should focus on controlling film growth, maintaining aptamer recognition activity, and validating the practical performance of composite interfaces in complex food matrices.

### 4.4. Novel Nanomaterials

The development of electrode-modification materials has gradually expanded to two-dimensional nanomaterials with high electrical conductivity, tunable surface chemistry, and unique layered structures. MXenes, a family of transition-metal carbides and/or nitrides generally represented by M_n+1_X_n_T_x_, have attracted particular interest in electrochemical sensing. Here, “M” denotes an early transition metal, “X” represents carbon or nitrogen, and “T_x_” denotes the surface terminals [117]. As an emerging two-dimensional layered nanomaterial, MXene exhibits metallic electrical conductivity, a high specific surface area, good hydrophilicity, and abundant surface functional groups (such as -O, -OH, and -F), offering a new material option for electrode modification [118,119]. For example, to achieve ultra-trace and accurate detection of Hg^2+^ in water samples, Li et al. [120] fabricated a three-dimensional porous MXene-supported AuNP composite by electrodeposition for mercury-ion detection. The electroactive surface area reached 0.0255 cm^2^, approximately three times that of the bare glassy carbon electrode, improving electron transfer and increasing the available sites for nucleic acid probe immobilization. Zhang et al. [118] prepared MXene-AuNP composites and combined them with a signal probe and catalytic hairpin assembly amplification strategy for tetracycline detection. The resulting sensor achieved an LOD of 0.789 pg/mL over a linear range of 0.001–500 ng/mL. These studies demonstrate how MXene-based interfaces can be combined with metal nanostructures and nucleic acid amplification to improve the overall sensing response.

Although MXenes exhibit excellent metallic conductivity, individual MXene flakes are prone to interlayer stacking and oxidative degradation during long-term operation. Specifically, in oxygen-containing aqueous electrolytes, they readily react with dissolved oxygen to form an insulating TiO_2_ passivation layer on their surface, impairing their intrinsic conductivity. Meanwhile, abundant polar terminal groups on the MXene surface render the flakes highly hydrophilic and van der Waals forces between the nanosheets induce spontaneous restacking and aggregation. This drastically reduces the effective electroactive surface area, blocks interlayer ion transport pathways, decreases exposed active sites, and ultimately lowers electrochemical reaction efficiency and compromises the structural stability of the overall framework [121]. To address these limitations, MXene can be hybridized with metal nanoparticles and carbon-based materials. On the one hand, external nanofillers can be intercalated into the interlayer spacing of MXene sheets, effectively expanding the layered structure, suppressing aggregation, and simultaneously enhancing the electrocatalytic activity of the system. On the other hand, the composite structure can effectively isolate MXene from direct contact with the aqueous environment, thereby significantly mitigating oxidative degradation. These synergistic effects enable the simultaneous improvement of electrode conductivity, effective electroactive surface area, and long-term sensing stability.

## 5. Applications of Electrochemical Aptasensors in Food Safety

In food contaminant detection, EABs can be coupled with large-scale analytical instruments such as high-performance liquid chromatography (HPLC) for sensitive and rapid screening of large batches of samples [58]. Although EABs use highly specific aptamers, their selectivity cannot fully eliminate matrix interferences from real food samples, which are rich in proteins, lipids, polysaccharides, salts, and electroactive metabolites. Proteins adsorb onto electrodes, forming insulating fouling layers that hinder electron transfer and suppress faradaic signals, while matrix–aptamer interactions alter conformations and reduce binding affinity, ultimately compromising repeatability and quantitative accuracy [122].

Sample pretreatment is the first line of defense against matrix interferences, removing interfering components before detection to improve the accuracy and reliability of electrochemical analysis. Common methods include dilution, centrifugation, filtration, protein precipitation, solvent extraction, and solid-phase extraction. The choice of method depends on matrix complexity: simple dilution or filtration often suffices for drinking water and fruit juices, while protein- and lipid-rich matrices such as milk, meat, and edible oils require protein removal, lipid extraction, and target purification. Effective pretreatment reduces non-specific electrode adsorption and matrix effects on aptamer binding affinity, enabling accurate and reliable quantification [123,124,125,126].

At the sensor level, further mitigation of matrix interferences can be achieved through antifouling electrode interfaces such as PEG, BSA, or MCH blocking layers, signal correction techniques including ratiometric sensing and internal standard baseline correction, and quantitative calibration using matrix-matched standard curves and the standard addition method. For simple diluted samples like fruit juice, matrix-matched calibration alone suffices; for complex protein and lipid-rich matrices, antifouling electrode modification combined with sample purification pretreatment is required [75,127]. With these challenges and mitigation strategies in mind, the following sections systematically review the recent advances of electrochemical aptamer-based biosensors (EABs), focusing on key detection targets including pesticide and veterinary drug residues, mycotoxins, heavy metal ions, and foodborne pathogenic bacterias…

### 5.1. Pesticide and Veterinary Drug Residues

Pesticide and veterinary drug residues constitute a major category of food contaminants. While these substances are essential for crop protection and disease prevention in livestock, their excessive or improper use inevitably leads to residues that accumulate through the food chain and pose potential risks to human health [128]. Therefore, the development of efficient, sensitive, rapid, and accurate methods for detecting pesticide and veterinary drug residues in food has become an urgent concern in the field of food safety. To address this issue, various EAB detection methods have been developed. Compared with natural enzymes [129] and antibodies [130] aptamers offer distinct advantages, including high specificity, broad target range, low-cost synthesis, and good stability. These properties make EABs a powerful platform for rapid and sensitive screening of such residues in food.

For protein-rich dairy matrices, sample dilution is a straightforward and effective approach to reduce matrix interference. Jackowska et al. [131] constructed an electrochemical sensor based on RNA aptamers, in which aptamers were immobilized via Au-S bonding for quantitative detection of neomycin (Neo) in milk. This method showed a linear range of 1–100 nM in 10-fold diluted milk, with an LOD as low as 36.3 nM. Notably, the aptasensor demonstrated significantly higher specificity toward neomycin than toward interfering antibiotics such as kanamycin, streptomycin, tetracycline, and oxytetracycline, confirming its good selectivity. Meanwhile, the 10-fold sample dilution effectively reduced the protein content in the milk matrix, and the sensor showed good recovery rates in spiked milk samples (89.3–96.1%), indicating acceptable anti-interference ability in dairy matrices. Hongoeb et al. [62] developed an aptasensor for highly sensitive detection of the herbicide GLY by immobilizing an amino-labeled aptamer onto 4-aminophenylacetic acid (CMA)-modified SPCE via amide bonding. The sensor exhibited a linear range of 0.01–10.0 μM, with an LOD as low as 0.0515 μM. Recovery rates in tap water, orange juice, and fresh strawberries ranged from 87.8–120.3%. A systematic *t*-test was conducted to validate the recovery results. The calculated *t*-values (ranging from 0.561 to 4.236) were lower than the critical value (4.303, *p* = 0.05), indicating no significant difference between the sensor results and theoretical spiked concentrations. These results demonstrate the high accuracy of the sensor and good anti-interference capability in real matrices, though the relatively wide recovery range suggests that matrix effects remain a challenge in more complex samples.

Highly sensitive detection in complex food matrices, such as meat and herbal samples, remains a significant challenge for EABs. To address this, researchers have adopted various strategies, including signal correction and nanomaterial-based signal amplification. In a representative study, Zhao et al. [132] developed a dual-signal ratiometric EAB for KAN detection using a Bio-Apt-TDN probe immobilized via Au–S bonding. Fe-NPC and MB@SA-COFs provided electrocatalytic amplification and enabled dual-signal output. The sensor achieved KAN detection in pork within 3 min, with an LOD of 0.4 pM and an RSD of 1.6%. For spiked milk samples, the results were comparable to those obtained by liquid chromatography–tandem mass spectrometry (LC–MS/MS), with RSDs below 5.0%. Notably, the sensitivity was two orders of magnitude higher than that of antigen–antibody-based electrochemical sensors [133]. This dual-signal ratiometric design thus exemplifies a typical signal correction strategy for enhancing detection accuracy in complex matrices. While signal correction strategies primarily improve accuracy by compensating for matrix-induced fluctuations to enhance accuracy, nanomaterial-modified interfaces focus on boosting sensitivity through signal amplification. Zhang et al. [134] developed an EAB for MAL detection in herbal samples using AgNPs@COFs prepared by room-temperature in situ reduction. The sensor showed a linear range of 10–800 ng/L and an LOD of 8.2 ng/L, improving sensitivity by three orders of magnitude compared with enzyme-based sensors [135]. Spiked recoveries of MAL ranged from 98.32% to 101.92% with RSDs of 1.25–3.28%, demonstrating good reliability for real-sample analysis. Taken together, these two strategies offer complementary strengths: the ratiometric dual-signal design primarily corrects matrix-induced fluctuations to ensure accuracy, whereas nanomaterial amplification boosts sensitivity through electrocatalytic enhancement. However, both rely on elaborate nanomaterial synthesis and multi-step electrode fabrication, which may limit their practical scalability.

### 5.2. Mycotoxin Residues

Mycotoxins are toxic secondary metabolites produced by fungi such as *Aspergillus*, *Fusarium*, and *Penicillium*, which commonly contaminate a wide range of food matrices including cereals, nuts, dried fruits, traditional Chinese medicines, and dairy products [136,137]. The most common types of mycotoxins in food include aflatoxin B_1_ (AFB_1_), ochratoxin A (OTA), deoxynivalenol (DON), fumonisin B_1_ (FB_1_), and zearalenone (ZEN). These compounds not only cause crop yield reduction and quality deterioration but also enter the human body through the food chain, inducing acute or chronic poisoning and even long-term carcinogenic and mutagenic effects, posing serious threats to public health [138]. In recent years, various mycotoxins have been detected in food, among which AFB_1_ and OTA have been the most extensively studied. Notably, mycotoxin-contaminated foods typically present complex matrices rich in proteins, lipids, polysaccharides, and pigments, which can cause significant non-specific adsorption and signal interference during electrochemical detection. This matrix effect poses a major challenge for the sensitive and accurate detection of mycotoxins, driving the development of high-performance EABs with improved anti-interference capabilities [139,140].

In AFB_1_ detection studies, Liu et al. [141] constructed an Apt/Au/N-CNFs/CFs working electrode for the detection of AFB_1_ in traditional Chinese medicine decoction pieces. The sensor exhibited a good linear response in the range of 10–1 × 10^8^ pg/mL, with an LOD as low as 6.4 pg/mL. The stability of the prepared EAB was evaluated by performing ten consecutive cyclic voltammetry scans, which yielded an RSD of 0.7%, indicating good signal reproducibility under repeated electrochemical measurements. To further overcome the limitation that single-signal modes are susceptible to environmental and matrix interference, Jiang et al. [142] developed a dual-mode EAB combining electrochemical quantification and visual colorimetry for AFB1 detection. The sensor relied on Apt-MB conformational changes after AFB_1_ binding to regulate electrochemical signals, while CoFe_2_O_4_@Au nanozyme catalyzed TMB oxidation for smartphone-assisted colorimetric analysis. The LODs were 0.34 pg/mL, 0.016 ng/mL, and 0.043 ng/mL for the electrochemical, colorimetric, and smartphone modes, respectively. The sensor showed good selectivity and achieved recoveries of 85.79–114.22% in peanut, corn, and wheat samples. Beyond signal correction strategies, engineering the signal transduction mechanism itself represents another direction to achieve high analytical sensitivity. Song et al. [143] developed a stimuli-responsive electrochemiluminescence (ECL) aptasensor for AFB_1_ detection using g-C_3_N_4_@Au as the luminophore and AuAgPtNSs as the ECL-RET quencher. Combined with DNAzyme-mediated signal regulation, the sensor achieved dual signal control with an LOD of 0.11 fg/mL and a wide linear range of 1 × 10^−9^–1 μg/mL. Alongside advances in signal engineering, the detection of other critical mycotoxins such as OTA has also attracted considerable attention. Hou et al. [144] constructed an OTA aptasensor by immobilizing OTA-apt on AuE through complementary nucleotide linkage. The sensor achieved an LOD of 0.05 ng/mL, with recoveries of 74.8–105.0% in malt samples, and its reliability was confirmed by ultra-fast liquid chromatography–tandem mass spectrometry (UFLC–MS/MS). To address the limitations of cDNA-mediated aptamer immobilization, such as aptamer entanglement, steric hindrance, and low recognition efficiency, Xu et al. [145] introduced a tetrahedral DNA nanostructure (TDN) as a rigid scaffold to overcome the limitations of cDNA-based aptamer immobilization. TDN was vertically assembled on AuE via Au-S bonding, enabling directional capture and optimized spatial distribution of OTA-apt, thereby reducing steric hindrance and nonspecific adsorption. The developed EAB achieved an LOD of 0.68 ng/L, with a 23-fold sensitivity improvement over cDNA-based sensors. Recoveries in wheat flour ranged from 89.1% to 105.4%, and results showed no significant difference from ultra-high-performance liquid chromatography (UPLC) analysis (*p* = 0.5457, *p* > 0.05), confirming its accuracy in real samples.

### 5.3. Heavy Metal Ion Residues

Heavy metals from industrial discharge, agricultural runoff, and geological sources can enter irrigation water, aquaculture systems, and agricultural soil, where they are taken up by crops and aquatic organisms and subsequently bioaccumulate along the food chain, posing threats such as multi-organ damage and chronic diseases [146,147]. While conventional large-instrument methods offer high sensitivity [147,148,149], they require complex sample pretreatment and bulky equipment, making them unsuitable for rapid on-site screening. EABs provide a promising alternative with fast response, high specificity, and ease of miniaturization. In this context, Monitoring heavy metals in environmental water and soil serves as an upstream safeguard for food safety, although direct detection in food commodities remains the ultimate goal. Heavy metal detection in real samples faces distinct matrix challenges. In aqueous matrices, coexisting metal ions and dissolved organic matter can interfere with aptamer binding, while in complex food matrices, proteins, polysaccharides, and minerals may cause non-specific adsorption and affect free ion concentration through complexation [150,151].

Among heavy metal contaminants, Hg^2+^ has been the most extensive analyte for EABs. Zhou et al. [152] developed an EAB for Hg^2+^ detection in river water, soil, and vegetable samples, using a black phosphorus–porous graphene (BP-PG) nanocomposite as the electrode substrate. Polylysine and AuNPs were introduced to enhance the immobilization efficiency of SH-apt. The sensor achieved an LOD of 0.045 nmol/L, far below the EPA limit for Hg^2+^ in drinking water (10 nmol/L), demonstrating its potential for environmental monitoring. To further improve sensitivity, Meng et al. [153] developed an EAB using an Fc-labeled Hg^2+^-apt as the recognition element. Upon Hg^2+^ binding, the aptamer formed a stable T-Hg^2+^-T hairpin structure, bringing Fc closer to the electrode and enhancing the electrochemical signal. The sensor exhibited a linear range of 0.01–100 nM with an LOD of 2.30 pM, enabling sensitive Hg^2+^ detection in seawater. Beyond Hg^2+^, Cd^2+^ represents another major heavy metal contaminant that inhibits biological growth, induces oxidative damage, and accumulates along the food chain. Xu et al. [154] developed an “off-on” ECL aptasensor for Cd^2+^ detection in Ganoderma lucidum based on an ECL-RET strategy. AIECL-active polymer dots were used as luminophore donors, while BHQ-labeled Cd^2+^-apt served as the recognition element. The sensor showed a linear range of 0.01–100 ppb with an LOD of 0.006 ppb. The results were consistent with ICP-MS measurements, with all RSDs below 8.7%, confirming its accuracy for Cd^2+^ detection. For more complex food matrices, dual-mode ratiometric sensing has been employed to improve detection accuracy. Tian et al. [155] constructed a PANI@Au conductive interface by electrochemical deposition and immobilized His-Pd-labeled Cd^2+^-apt on the electrode. A dual-mode aptasensor integrating ratiometric electrochemical and colorimetric signals was developed for Cd^2+^ detection. The LODs reached 3.40 × 10^−2^ ng/L and 3.10 × 10^−2^ ng/L for the electrochemical and colorimetric modes, respectively. The sensor achieved recoveries of 94.49–104.67% in fish, shrimp, and scallop samples. These results demonstrate that dual-mode ratiometric EABs can achieve reliable heavy metal quantification in animal-derived food matrices with complex compositions.

In addition to Hg^2+^ and Cd^2+^, EABs have also been developed for the detection of other common heavy metal contaminants such as As^3+^ and Pb^2+^ [156,157]. Baghbaderani et al. [63] developed an EAB for As^3+^ detection using a chitosan–Nafion conductive platform coupled with CNT-BSA signal amplification. The sensor achieved an LOD of 74 pM and a sensitivity of 100.82 Ω·nM^−1^. Recoveries in tap water and industrial wastewater samples were consistent with HG-AAS results, with RSDs below 6.6%, confirming its accuracy and reliability for rapid As^3+^ detection in real water samples. Zhang et al. [158] designed an S1/AuNPs/Ti_3_C_2_-MXene/GCE sensing interface for highly sensitive detection of Pb^2+^ in water. The sensor achieved an LOD as low as 3 × 10^−7^ M, with a detection time of only 25 min. Recoveries in tap water and river water ranged from 96.97% to 108.03%. Nevertheless, both the As^3+^ and Pb^2+^ sensors described above were validated exclusively in environmental water samples, and their performance in food matrices remains unverified. Given that seafood, rice, and vegetables are major dietary sources of As^3+^ and Pb^2+^ exposure, adapting these immobilization and signal amplification strategies to such food commodities represents a worthwhile direction for future research.

### 5.4. Detection of Foodborne Pathogenic Bacteria

Foodborne pathogenic bacteria are major threats to food safety and public health, potentially causing outbreaks [159]. Common pathogenic bacteria in food mainly include *Escherichia coli* (*E. coli*), *Salmonella typhimurium* (*S. typhimurium*), *Listeria monocytogenes* (*L. monocytogenes*), and *Staphylococcus aureus* (*S. aureus*), which can enter the food chain through contaminated raw materials and processed foods, posing risks to human health [160]. Notably, the detection of foodborne pathogens in real samples is often challenged by matrix interference from food components such as proteins, fats, and sugars. Therefore, the development of rapid and accurate detection methods for foodborne pathogenic bacteria is of great importance.

For relatively simple liquid food matrices, Sheikhzadeh et al. [61] used poly[pyrrole-co-3-carboxyl-pyrrole] as the sensing interface to construct an EAB for the detection of *S. typhimurium* in apple juice. The method showed a good linear response in the range of 10^2^–10^8^ CFU/mL, with an LOD as low as 3 CFU/mL. To further improve sensitivity, signal amplification strategies have been employed. Zhang et al. [161] developed an EAB for ultrasensitive detection of *E. coli* based on a Zn^2+^-triggered DNAzyme-driven swing-arm walking system and a COF@Au-Thi/H_4_ multifunctional signal probe. The sensor employed a triple-helix molecular switch (THMS); Upon target recognition, THMS released the S2 primer to initiate strand displacement amplification, while Zn^2+^-activated DNAzyme further enhanced the signal through cyclic substrate cleavage. The amplified electrochemical response enabled an LOD of 10 CFU/mL, with recoveries of 89.5–110.5% in fruit juice and milk samples. For complex food matrices requiring multi-target detection, Chi et al. [162] developed a multiplex EAB for simultaneous detection of *S. aureus*, *E. coli*, and *S. typhimurium* using MWCNT-COOH/MOF-818 as the sensing interface. Three aptamers were immobilized through thiol-ene click chemistry, achieving LODs of 3, 6, and 4 CFU/mL, respectively, within 8 min. The sensor showed recoveries of 89.3–127% in pork paste samples, with results consistent with the plate count method. This multiplex strategy enables rapid and sensitive on-site screening of foodborne pathogens in complex matrices.

From the perspective of technological and conceptual evolution, recent advances in aptamer sensors represent a profound paradigm shift from conventional, single-mode detection methods toward highly integrated sensing strategies. Rather than merely applying newly screened aptamers to traditional transducers, recent technological developments focus on the synergistic optimization of four core conceptual pillars: (i) rational manipulation of target-induced aptamer conformational changes to improve binding kinetics and specificity; (ii) integration of versatile signal amplification cascades for ultrasensitive detection; (iii) precise interfacial engineering to minimize nonspecific adsorption in complex matrices; and (iv) the design of multifunctional electrode materials that simultaneously boost conductivity, catalytic activity, and biocompatibility. Despite these remarkable technological breakthroughs, critical challenges regarding sensor reproducibility, long-term stability, and large-scale fabrication remain to be fully addressed for practical point-of-care applications.

Table 2 presents a summary of recent advances in EABs for the detection of various food hazards.

## 6. Conclusions and Perspectives

This review systematically summarizes recent advances in EABs for food safety detection. By synergistically combining the molecular specificity of aptamers with the high sensitivity, rapid response, and miniaturization potential of electrochemical transduction, EABs have emerged as powerful platforms for screening trace contaminants in complex food matrices. Across potentiometric (simple instrumentation), voltammetric (versatile CV characterization and sensitive DPV/SWV quantification), and impedimetric (label-free resistance tracking) modalities, recent conceptual and technological breakthroughs have evolved along five main dimensions: (i) innovative readout designs, utilizing internal-reference ratiometric probes (e.g., hairpin-tetrahedral DNA nanostructures and MB-cDNA architectures) and smartphone-based electrochemical/colorimetric dual-mode platforms for self-calibrating cross-validation; (ii) advanced signal amplification, driven by catalytic hairpin assembly, exonuclease-assisted recycling, and nanozyme catalysis; (iii) rational interface engineering, shifting from single components to multifunctional MXene/MOF/COF composites and rMoS_2_–Au heterointerfaces that synergize high conductivity, large surface area, and rapid electron transfer; (iv) tailored aptamer immobilization via covalent bonding, Au–S self-assembly, streptavidin–biotin affinity, and complementary hybridization, notably advancing toward oriented cDNA scaffolds that integrate anchoring with signal transduction; and (v) broadened real-sample applications targeting agrochemical residues, mycotoxins, heavy metals, and pathogens using multiplexed arrays and portable screen-printed/paper-based electrodes.

Despite this remarkable progress, translating EABs from laboratory prototypes into commercial on-site tools still faces critical bottlenecks that require targeted future efforts: (i) To overcome non-specific adsorption and matrix-induced signal fluctuations in complex food samples, future designs must integrate antifouling interfaces, ratiometric/dual-mode self-correction, matrix-matched calibration, and standardized sample pretreatment to ensure quantitative accuracy. (ii) Given the inherent trade-offs among stability, orientation control, fabrication complexity, and regenerability across the four immobilization strategies, more efforts should be directed toward rigid, oriented anchoring scaffolds (e.g., tetrahedral DNA nanostructures) alongside simplified, reproducible immobilization protocols that preserve aptamer bioactivity. (iii) To address the aggregation, oxidative degradation, complex synthesis, and high cost of current nanomaterials (metals, nanocarbons, conducting polymers, and MXenes), future interface engineering should prioritize low-cost, scalable, and environmentally stable composites that balance signal amplification with fabrication simplicity. (iv) Moving beyond single-analyte laboratory validation requires integrating multiplexed sensing arrays with pocket-sized electrochemical readers to achieve consistent field performance across diverse food matrices. (v) Continuous innovations in high-throughput aptamer selection and post-SELEX sequence optimization will remain vital for enhancing binding affinity under harsh matrix conditions. Ultimately, future EAB development should be driven by resolving these practical engineering bottlenecks–matrix antifouling, immobilization stability, material scalability, and field validation–rather than pursuing ultralow sensitivity alone, thereby paving the way for practical food safety monitoring and industrial translation.

## Figures and Tables

**Table 1:** Effects of different immobilization strategies on the performance of electrochemical aptasensors.

Methods	Immobilization Method	Advantages	Disadvantages
Covalent binding	Aptamers are immobilized through covalent bonds formed between functional groups (e.g., amino, carboxyl, thiol groups) on the electrode surface and aptamer molecules [71,72,73]	High binding strength, low aptamer detachment, and good interfacial stability and reproducibility [74]	Surface modification involves complicated steps and tends to leave residual chemical reagents inducing interfacial interference [75]
Self-assembly	Thiolated aptamers spontaneously assemble on gold surfaces through Au–S interactions to form ordered monolayers [76]	Simple operation, most widely used method, enhanced sensitivity and interfacial controllability [75]	Prone to non-specific adsorption, thereby increasing interfacial interference and affecting the detection signal [75]
Affinity interaction between streptavidin and biotin	Biotin-labeled aptamers are immobilized through the high-affinity interaction between biotin and streptavidin [77]	High affinity ensuring firm aptamer attachment, combined with multivalent binding capability that raises aptamer surface density, thereby boosting sensor sensitivity [77]	EDC/NHS crosslinking increases stability at the expense of higher complexity and cost, hindering large-scale application [78]
Complementary nucleotide chain linkage	Aptamers are immobilized through hybridization with complementary DNA strands previously attached to the electrode surface [70]	Base-pairing-mediated oriented aptamer anchoring, broad versatility, and suitability for a wide range of sensing configurations [70]	Complex annealing/hybridization procedures, reduced immobilization efficiency from electrostatic repulsion and steric hindrance, and need for extra optimization of system physicochemical properties [70]

**Table 2:** Application of electrochemical aptasensors in food safety detection.

	Analytes	Electrode Materials	Immobilization Methods	Analytical Methods	Linear Range (ng/mL)	LOD (ng/mL)	Recovery (%)	Stability (d)	Real Samples	Ref.
Pesticides and veterinary drugs	NEO	/	Self-assembly	EIS	0.61–61.50	12.0	89.3–96.1	4	Milk	[131]
	GLY	CMA	Covalent binding	EIS	1.69–1690.7	8.71	87.8–120.3	7	Tap water, orange Juice, strawberries	[62]
	KAN	AuNPs/Fe-NPC, MB@SA-COFs	Self-assembly	SWV	4.85 × 10^−4^–484.5	1.94 × 10^−4^	98.9–103.1	10	Milk	[132]
	MAL	AgNPs@COFs	Self-assembly	DPV	0.01–0.8	8.2 × 10^−3^	98.32–101.92	/	*Panax ginseng*, *Astragalus membranaceus*, *Lycium barbarum*, *Lonicera japonica*	[134]
Mycotoxins	AFB_1_	Au/N-CNFs/CFs	Self-assembly	DPV	0.01–1.0 × 10^5^	6.4 × 10^−3^	96.18–112.87	/	Chinese Materia Medica decoction pieces	[141]
	AFB_1_	CoFe_2_O_4_@Au	Self-assembly	SWV	1 × 10^−3^–100	3.4 × 10^−4^	85.79–113.78	/	Peanut, corn, wheat	[142]
	AFB_1_	AuAgPt NFs/g-C_3_N_4_@Au	Complementary nucleotide chain linkage	ECL	1 × 10^−6^–1 × 10^3^	1.1 × 10^−7^	95.4–105.3	15	Peanut, corn, rice	[143]
	OTA	/	Complementary nucleotide chain linkage	EIS	0.05–10	0.05	74.8–105	/	Malt	[144]
	OTA	/	Complementary nucleotide chain linkage	DPV	1 × 10^−3^–8.0	6.8 × 10^−4^	89.1–105.4	21	Wheat flour	[145]
Heavy metals	Hg^2+^	AuNPs/PLL/BP-PG	Self-assembly	DPV	0.2–2.0 × 10^3^	9.0 × 10^−3^	93.68–102.43	15	River water, soil, vegetable	[152]
	Hg^2+^	Au/Ag/PEDOT	Self-assembly	DPV	2.0 × 10^−3^–20.0	4.61 × 10^−4^	/	35	Seawater	[153]
	Cd^2+^	Pdots	Complementary nucleotide chain linkage	ECL	0.01–100	6.0 × 10^−3^	/	/	*Ganoderma lucidum*	[154]
	Cd^2+^	PANI@Au	Self-assembly	DPV	0.01–1 × 10^5^	3.40 × 10^−5^	94.49–104.67	25	Fish, shrimp, scallop	[155]
	As^3+^	Chit-Naf	Complementary nucleotide chain linkage	EIS	1.12 × 10^−2^–0.75, 1.5–1.87	5.54 × 10^−3^	92.36–118.50	/	Drinking water, industrial wastewater	[63]
	Pb^2+^	AuNPs/Ti_3_C_2_-MXene	Self-assembly	SWV	1.04–6.22 × 10^4^	6.22 × 10^−2^	96.97–108.03	7	Tap water, river water	[158]
Foodborne pathogenic bacterias	*S. typhimurium*	poly[pyrrole-co-3-carboxyl-pyrrole]	Covalent binding	EIS	10^2^–10^8^ CFU/mL	3 CFU/mL	/	/	Apple juice	[61]
	*E. coli*	PDDA-Gr/AuAgNSs	Complementary nucleotide chain linkage	DPV	10–10^8^ CFU/mL	10 CFU/mL	89.50–110.50	20	Fruit juice, milk, human serum	[161]
	*S. aureus*, *E. coli*, *S. typhimurium*	MWCNT-COOH/MOF-818	Covalent binding	0.2218	10–10^8^ CFU/mL	3 CFU/mL, 6 CFU/mL, 4 CFU/mL	89.30–127.00	20	Pork paste	[162]

## Data Availability

No new data were created or analyzed in this study. Data sharing is not applicable to this article.
